# Chemical Composition, Quality, and Bioactivity of *Laurus nobilis* L. Hydrosols from the Adriatic Regions of Croatia: Implications for Dermatological Applications

**DOI:** 10.3390/antiox14060688

**Published:** 2025-06-05

**Authors:** Lea Juretić, Valerija Dunkić, Ivana Gobin, Suzana Inić, Dario Kremer, Marija Nazlić, Lea Pollak, Silvestar Mežnarić, Ana Barbarić, Renata Jurišić Grubešić

**Affiliations:** 1 Faculty of Medicine, University of Rijeka, 51000 Rijeka, Croatia; lea.juretic@medri.uniri.hr (L.J.); ivana.gobin@medri.uniri.hr (I.G.); silvestar.meznaric@medri.uniri.hr (S.M.); ana.planinic@fzs.sum.ba (A.B.); 2 Faculty of Science, University of Split, 21000 Split, Croatia; dunkic@pmfst.hr (V.D.); mnazlic@pmfst.hr (M.N.); 3 Teaching Institute of Public Health of Primorje-Gorski Kotar County, 51000 Rijeka, Croatia; 4 Faculty of Pharmacy and Biochemistry, University of Zagreb, 10000 Zagreb, Croatia; suzana.inic@pharma.unizg.hr; 5 Faculty of Agriculture, University of Zagreb, 10000 Zagreb, Croatia; dkremer@agr.hr; 6 Croatian Institute of Public Health, 10000 Zagreb, Croatia; lea.pollak@hzjz.hr; 7 Faculty of Health Studies, University of Mostar, 88000 Mostar, Bosnia and Herzegovina

**Keywords:** antioxidant capacity, antimicrobial activity, gas chromatography-mass spectrometry, *Laurus nobilis* hydrosol, microwave extraction, quality, polyphenols, volatile compounds, wound-healing assay

## Abstract

*Laurus nobilis* L., Lauraceae, bay laurel, has been traditionally used for its various therapeutic properties, and in recent years has been gaining interest for its potential applications in skincare products. However, the biological effects of bay laurel, particularly its hydrosols, a water fraction obtained during essential oil production, remain unexplored. The objective of this study was to identify the volatile compounds in *L. nobilis* hydrosols (LnHYs) from different coastal regions of Croatia (north, middle, and south Adriatic) and to evaluate their potential safety and efficacy for dermatological applications. Upon isolating LnHYs using microwave-assisted extraction, LnHY volatiles were identified and quantified using gas chromatography and mass spectrometry. Oxygenated monoterpenes were the dominant compounds in all LnHYs (61.72–97.00%), with 1,8-cineole being the most abundant component (52.25–81.89%). The physical and chemical parameters of LnHYs were investigated to assess their purity and quality. Biological activity (cytotoxicity and wound-healing effect) was tested on the human keratinocyte cell line (HaCaT), selected as the experimental model due to its relevance to skin biology. Additionally, contents of polyphenolic substances, antioxidative effects using the Oxygen Radical Absorbance Capacity (ORAC) and 2,2-diphenyl-1-picrylhydrazyl (DPPH) methods, and the antimicrobial activity of LnHYs toward five skin microorganisms were determined. All tested hydrosols showed similar biological activity, with only minor differences. Cytotoxicity studies indicated the safety of the dermatological application of LnHYs, and the results of the wound-healing assay showed their neutral to mildly positive effect. Considering the growing use of bay laurel preparations in pharmaceutical and cosmetic applications, extensive studies on their biological activity, quality, and safety are essential to either support or regulate their use in humans.

## 1. Introduction

The Croatian flora is one of the richest in Europe, with over 5000 different plants [1]. Many medicinal plant species of the Croatian Adriatic region have been in traditional use since ancient times, one of which is bay laurel (*Laurus nobilis* L., Lauraceae). Native to southern Europe and the Mediterranean region, bay laurel is widespread along the Croatian Adriatic coast. It is an aromatic evergreen tree or large shrub, widely used in both culinary practices and traditional medicine. The bay leaf (Lauri folium) is the most commonly used part of the plant, though the fruit is also utilized [2].

Bay laurel has been traditionally employed to treat various health conditions, including dermatological, neurological, rheumatic, gastrointestinal, and respiratory disorders. It is also used as an antiseptic, immunostimulant, expectorant, analgesic, and antispasmodic and for gastrointestinal problems. However, scientific evidence on its phytotherapeutic efficacy in these health conditions is largely lacking [3].

Previous studies on the bioactive effects of bay laurel have shown that leaf preparations have antioxidant, neuroprotective, and anticholinergic properties [4,5]. The antioxidant activity of bay leaf essential oil (EO), ethanol extract, and decoction has been tested and confirmed by the 2,2-diphenyl-1-picrylhydrazyl (DPPH) method and the β-carotene method [4]. Furthermore, studies of bay leaf alcohol extract have been proven with several different methods to have neuroprotective and antioxidant activity [5]. EO and other preparations of bay laurel from around Europe have been tested for biological effects, such as antibacterial, antifungal, and antioxidant activity [6,7,8,9,10,11]. However, hydrosols—the water fraction of the essential oil extraction process—have been insufficiently researched. Hydrosols, also known as hydrolates, are aqueous distillates obtained as co-products during the steam distillation of aromatic plants for essential oil extraction. These aromatic waters contain water-soluble compounds and trace amounts of volatile constituents, making them distinct from their essential oil counterparts. Hydrosols, commonly regarded as waste products of essential oil extraction, have nevertheless been found to contain a wealth of biologically active compounds [12]. Hydrosols derived from aromatic plants are particularly interesting due to their rich chemical composition and diverse biological activities, and they have gained attention for their potential applications in aromatherapy, cosmetics, food preservation, and medicine [2,6].

While EOs have strong antimicrobial effects, hydrosols offer gentler alternatives, making them increasingly popular in skincare and therapeutic applications [6].

This study focuses on the green extraction (microwave-assisted extraction, MAE) of bay laurel leaves and an evaluation of the potential applications of *L. nobilis* hydrosols (LnHYs) on human skin. The isolation of free volatile compounds, as important plant metabolites, can be achieved through both classical and green extraction methods. Classical extraction techniques include steam distillation, hydrodiffusion, hydrodistillation, destructive distillation, and cold pressing, whereas green extraction methods comprise turbo distillation, ultrasound-assisted extraction, microwave-assisted extraction, and instant controlled pressure drop (DIC) technology. The composition of the EO extracted from the same plant material can vary depending on the isolation technique, as factors such as distillation duration, temperature, pressure, and plant material quality play a crucial role. Compared to traditional methods, green extraction requires less time and water [13].

To explore possible regional influences, we analysed hydrosols obtained by MAE from locations in three regions of Croatia: north, middle, and south Adriatic. Our objectives encompassed a comprehensive phytochemical analysis of LnHYs, focusing on volatile compounds and leaf polyphenolic content. Additionally, we assessed quality parameters and examined biological activities, including antioxidant capacity and antimicrobial effects against common dermatological pathogens. We also evaluated the cytotoxicity of LnHYs on the human keratinocyte cell line (HaCaT) to determine potential toxicity to dermal cells, along with their effects on wound healing using the scratch assay.

Most of the chemical, biological, and quality characteristics of laurel hydrosols examined in this study have not been previously researched, and the results are presented here for the first time. This study assesses the phytotherapeutic potential of LnHYs from various Croatian coastal regions by comparing their chemical composition and biological activity, supporting their possible dermatological applications. By expanding the knowledge of regional influences on the composition of LnHYs—and emphasising their potential applications—we aim to enhance the scientific understanding of hydrosols while promoting sustainable practices in industries that rely on natural ingredients.

## 2. Materials and Methods

### 2.1. Plant Material

*L. nobilis* leaves were collected in August 2022 in Adriatic regions of Croatia from three distinct locations labelled as north, middle, and south Adriatic. The samples were collected at the following locations: Lovran (45°28′7″ N, 14°27′25″ E), elevation 10 m as the north sample; Tisno (43°47′59″ N, 15°38′32″ E), elevation 10 m as the middle sample; and Korčula (42°56′24″ N, 16°47′26″ E), elevation 50 m as the south sample. The aim was to collect a representative sample of plant material from different locations along the Adriatic coast and islands that differ in both geographical and climatic characteristics.

The plant material consisted of healthy, fully developed bay leaves, without visible mechanical or biological damage. The bay leaves were harvested from about ten different trees at each location of the three Adriatic regions.

Plant material was air-dried in a single layer, protected from direct sunlight, for ten days. Once dried, the bay leaves were stored in double-sided paper bags, properly labelled, and kept in a dry, dark place until analysis.

Voucher specimens were deposited in the Herbarium of the Botany Laboratory, Faculty of Science, University of Split, Croatia (Voucher Nos: CROLn-01-2022, CROLn-02-2022, and CROLn-03-2022).

### 2.2. Isolation of Hydrosols

Part of the dried plant material was subjected to the microwave extraction procedure (green isolation) at the Faculty of Science, University of Split, using a Milestone ETHOS X Microwave Laboratory Oven microwave extraction device (Milestone S.r.l., Sorisole (BG), Italy). Essential oil (EO) and hydrosols from the bay leaves were isolated by microwave extraction, which is more energy-efficient and environmentally friendly compared to classical methods of EO isolation [14].

Dried bay leaves (140 g) from each laurel population were subjected to microwave-assisted hydrodistillation using an ETHOS X extraction system (Milestone, Italy). The extraction was carried out at atmospheric pressure for 30 min, following a 10 min preheating phase at 800 W (98 °C). The resulting distillates separated into two distinct layers: a lipophilic essential oil phase and an aqueous hydrosol phase (LnHY). The prepared hydrosol samples were stored in well-sealed dark glass bottles at 4 °C until analysis. A 2 g aliquot of each LnHY sample was transferred into a sealed glass vial. For volatile compound extraction, vials were placed in a water bath, and a solid-phase microextraction (SPME) needle was inserted through the septum. The headspace was equilibrated at 40 °C for 20 min, followed by stirring for an additional 20 min to allow the adsorption of volatiles onto the SPME fibre. The fibre was then inserted into the injection port of a gas chromatograph and held there for 20 min to enable the desorption of the volatile compounds [14,15,16].

### 2.3. Identification of Hydrosol Components

The analysis of hydrosol components was carried out using a Varian 3900 gas chromatograph (model 3900; Varian Inc., Lake Forest, CA, USA) equipped with both a flame ionisation detector (FID) and a 2100T mass spectrometer (Varian Inc., Lake Forest, CA, USA). Two types of columns were employed: a non-polar VF-5 ms capillary column (30 m × 0.25 mm i.d., 0.25 µm film thickness, Palo Alto, CA, USA) and a polar CP Wax 52 column (30 m × 0.25 mm i.d., 0.25 µm film thickness, Palo Alto, CA, USA). For the HY analysis, the FID was maintained at 300 °C and the injector at 250 °C, with helium as the carrier gas at a flow rate of 1 mL/min. The VF-5 ms column temperature was held isothermally at 60 °C for 3 min then increased at a rate of 3 °C/min to 246 °C and maintained at that temperature for 25 min. For the CP Wax 52 column, the temperature was held at 70 °C for 5 min then ramped at 3 °C/min to 240 °C and held for 25 min. A 2 µL injection volume was used with a 1:20 split ratio. Mass spectrometry conditions included an ion source temperature of 200 °C, an ionisation voltage of 70 eV, and a mass scan range of 40–350 *m*/*z* [14,17,18]. Peak identification was based on the comparison of the retention indices of n-alkanes with those of reference standards, previous studies [19], in-house libraries, and published literature. The results are presented as the mean of three replicates with standard deviation.

### 2.4. Physical and Chemical Parameters

A standard buffer solution at pH 4.00 and pH 7.00, ethanol (96%), sodium chloride (NaCl), potassium hydroxide (KOH, 0.1 M), and phenolphthalein (1% ethanol solution) were purchased from Kemika (Zagreb, Croatia). All reagents and chemicals were of analytical grade. Weighing was performed using a Mettler Toledo XP 205 analytical balance (Mettler-Toledo GmbH, Gießen, Germany) with an accuracy of 0.01 mg. A Mettler Toledo MP 220 pH meter (Mettler-Toledo GmbH, Germany) with a resolution of 0.01 was used to measure the pH of hydrosols, calibrated using pH 4.00 and 7.00 buffers.

The relative density of EO is the ratio between its volume mass and the volume mass of the reference compound (water at 20 °C).

LnHY relative density (*dr*) was determined by using a pycnometer at 20 °C and calculated as shown in Equation (1):

*dr* (LnHY) = *d* (LnHY)/*d* (distill. H_2_0) = p2 − p/p1 − p (1)

where *d* is the mass density, p and p1 are the mass of the dry pycnometer and pycnometer with distilled water, respectively, and p2 is the mass of the pycnometer with the sample, all expressed in grams.

A Hanon A670 automatic refractometer (Hong Kong, China) with a resolution of up to 0.00001 (nD) was used to determine the refractive index (*n*) of hydrosols at 20 °C (λ = 589.3 nm). Calibration was performed with distilled water (*n* = 1.3329). The refractive index is defined as the ratio between the sine of the angle of incidence and the sine of the angle of refraction of a luminous ray of a predetermined wavelength in the EO maintained at a constant temperature [19].

Turbidity was measured using a Hach 2100P turbidimeter (Hach Company, Loveland, CO, USA) and expressed in Nephelometric Turbidity Units (NTUs).

The acid value (*AV*), representing the mass of KOH (in mg) required to neutralise free acids in 1 g of the sample, was determined by titration. An aliquot of 1.00 mL hydrosol was mixed with 30 mL of 96% ethanol and a phenolphthalein indicator and then titrated with 0.1 M KOH. The acid value was calculated based on the volume of KOH (in mL) using Equation (2):

*AV* = 5.610 × V0.1 M KOH/m(LnHY) (2)

The essential oil content (%) in the hydrosol samples was determined by water distillation (90 min) using an Unger apparatus and calculated according to Equation (3):

essential oil (%) = V (essential oil) (mL)/V(LnHY) (mL) × 100(3)

### 2.5. Polyphenol Analysis

#### 2.5.1. Apparatus and Chemicals

All absorbance measurements were performed using a water bath, a reflux condenser, and a UV/Vis spectrophotometer (Agilent 8453, Agilent, Karlsruhe, Germany) with a PC-HP 845x UV-Visible System (Agilent, Karlsruhe, Germany) and 1 cm quartz cells. Sample solutions were filtered using a 0.20 μm Minisart-plus membrane filter (Sartorius AG, Göttingen, Germany) [20].

*Pro analysi* chemicals and double-distilled water were used throughout the study. A 30% methanol solution was used for the extraction of plant material. Sodium carbonate decahydrate (33%) was used for sample preparation. Folin–Ciocalteu phenol reagent (FCR) for spectrophotometric analysis and casein (tannin precipitation) were supplied by Merck (Darmstadt, Germany), standard flavonol quercetin by Roth (Karlsruhe, Germany), and other chemicals and reagents for polyphenol analysis were of analytical grade and supplied by Kemika (Zagreb, Croatia).

A polyphenol analysis was conducted on the laurel leaf samples from the three selected locations (north, middle, and south Adriatic) using three different spectrophotometric methods (FCR assay, TF assay, and TPA assay, see below).

#### 2.5.2. Total Polyphenol and Tannin Analysis (FCR Assay)

The total polyphenols (TPs) and tannins (Ts) in the *L. nobilis* leaves were determined using the prevalidated FCR assay procedure for polyphenol analysis [21] as follows:

*Extraction of polyphenols and tannins:* Leaves were ground (0.250 g) and extracted with 30% methanol, followed by filtering and dilution to prepare a solution (S1).

*Separation of tannins:* The solution S1 was then treated with casein to separate tannins. The resulting filtrate (S2) was used to isolate tannins specifically bound to casein.

*FCR assay:* Both S1 (for total polyphenols) and S2 (for tannins bound to casein) were analysed using the Folin–Ciocalteu reagent (FCR) and sodium carbonate (33%) to measure absorbance at 720 nm. The absorbance values correlate with the concentration of polyphenols and tannins in the extracts. A calibration curve was created using tannin as a standard to calculate the tannin content in the extracts. The difference in the absorbance of S1 and S2 reflects the tannins precipitated with casein, while the absorbance of S1 represents the total polyphenols (TPs).

The contents of TPs and Ts in the *L. nobilis* leaves were evaluated through three independent analyses and expressed as a percentage of the dry weight of the herbal material, according to Equations (4) and (5):

TP (%) = AS1/0.0025(4)

T (%) = AS1/0.0025 − AS2/0.0025(5)

where AS1 and AS2 represent the measured absorbance values of samples S1 and S2, respectively.

#### 2.5.3. Total Flavonoid Analysis (TF Assay)

The TF assay describes the quantitative analysis of total flavonoids in bay laurel leaves using a spectrophotometric method where flavonoids are quantified as quercetin equivalents. Complexation of flavonoids with aluminium chloride enables specific detection at 425 nm [22].

The key steps of the TF assay are as follows:

*Extraction:* 0.200 g powdered herbal drug was extracted with acetone, hydrochloric acid, and hexamethylenetetramine under reflux for 30 min. The residue was re-extracted three times with acetone, and the filtrates were combined and diluted to 100.0 mL.

*Liquid–liquid extraction:* 20 mL hydrolysate was mixed with water and extracted with ethyl acetate in multiple steps. The ethyl acetate phases were washed with water, filtered, and diluted to 50.0 mL.

*Complex formation and spectrophotometry:* Two 10.0 mL portions of the extract were prepared, one with aluminium chloride and the other without a reagent (compensation solution). The absorbance was measured at 425 nm after 45 min.

*Calculation of flavonoid content:* The total flavonoid content (TF%) was calculated as quercetin equivalents using the following Equation (6):

TF (%) = A × 0.772/b(6)

where A is the absorbance and b is the drug weight (g).

#### 2.5.4. Total Phenolic Acid Analysis (TPA Assay)

The TPA assay was conducted on laurel leaf samples using the spectrophotometric method prescribed by the European Pharmacopoeia [22] as follows:

*Preparation of stock solution:* 0.200 g powdered herbal drug was mixed with 80 mL 50% ethanol and heated in a reflux flask in a boiling water bath for 30 min. After cooling and filtration, the filtrate was transferred to a 100 mL volumetric flask and diluted to volume with 50% ethanol, forming the stock solution.

*Preparation of test solution:* 1.0 mL stock solution was mixed with 2.0 mL 0.5 M hydrochloric acid, 2.0 mL solution containing 10 g sodium nitrite and 10 g sodium molybdate dissolved in 100.0 mL water, and 2.0 mL 8.5% sodium hydroxide solution. The flask was then filled to 10.0 mL with distilled water, forming the test solution.

*Preparation of compensating solution:* A compensating solution was prepared by diluting 1.0 mL stock solution with distilled water to a final volume of 10.0 mL.

*Absorbance measurement:* The absorbance of the test solution was immediately measured at two wavelengths: 505 nm (for rosmarinic acid) and 525 nm (for chlorogenic acid).

*Calculation of TPA content:* The mass fraction (%) of phenolic acids was calculated and expressed as rosmarinic acid (TPA1) using Equation (7):

TPA1 (%) = A × 2.5/m(7)

where A is the measured absorbance at 505 nm, taking the specific absorbance of rosmarinic acid at 505 nm to be 400, and m is the drug weight (g).

The mass fraction of phenolic acids was calculated and expressed as chlorogenic acid (TPA2) using Equation (8):

TPA2 (%) = A × 5.3/m(8)

where A is the measured absorbance at 525 nm, taking the specific absorbance of chlorogenic acid at 525 nm to be 188, and m is the drug weight (g).

### 2.6. Antioxidant Capacity of Hydrosols

#### 2.6.1. Measurement of the Oxygen Radical Absorbance Capacity (ORAC) Values

The assay was performed using a Tecan Infinite 200 PRO spectrophotometer (Tecan Trading AG, Zurich, Switzerland) and 96-well black polystyrene microtiter plates (Porvair Sciences, Leatherhead, UK). Each reaction mixture consisted of 180 µL fluorescein (1 µM), 70 µL 2,2′-azobis(2-methyl-propionamidine) dihydrochloride (AAPH, 300 mM; Acros Organics, Geel, Belgium), and 30 µL of either a blank (water, as hydrosols are aqueous extracts), a plant extract, or the reference standard Trolox (6.25–50 µM; Sigma-Aldrich, St. Louis, MO, USA). All plant extract solutions were diluted in phosphate buffer (0.075 mM, pH 7.0). Volatile compounds from each hydrosol were extracted and quantified, with concentrations expressed as μg volatiles per mL hydrosol. The test samples used for the ORAC analysis were diluted 80-fold and 160-fold. ORAC values for the hydrosols were reported as µmol Trolox equivalents (TE) per gram of extracted volatile compounds. The results represent the mean of three independent experiments.

#### 2.6.2. Measurement of the 2,2-Diphenyl-1-Picrylhydrazyl (DPPH) Radical Scavenging Activity

The DPPH method used to assess the antioxidant capacity of the extracts in this study was previously described by Mensor et al. [20] and Payet et al. [23] and was adapted for the plant extracts analysed. The procedure was carried out using a Tecan Infinite 200 PRO spectrophotometer (Tecan Trading AG, Zurich, Switzerland) and 96-well transparent polystyrene microtitre plates (Porvair Sciences, Leatherhead, UK). A volume of 100 µL methanol (Kemika, Zagreb, Croatia) was added to each well, with the exception of the first row, which contained either samples or standards. The plant extracts were prepared as described for the ORAC method. In the first wells, 200 µL undiluted hydrosol was added. Serial dilutions of the samples and Trolox were performed by transferring 100 µL from the first row to the second using a multichannel pipette. This process was repeated down the plate, with 100 µL discarded from the final row after mixing. A blank (water) was always placed in the first column of the 96-well plate, Trolox was placed in the second column, and the remaining columns were filled with the plant extracts. The reaction was initiated by adding 100 µL 200 µM DPPH methanol solution to each well. The initial absorbance at 517 nm was measured immediately, with an expected value of approximately 1.1. After 30 min of incubation, the absorbance was remeasured, and the percentage of DPPH inhibition was calculated using the following formula, as described by Yen and Duh [24], Equation (9):

% inhibition = [(AC_(0)_ − AA_(t)_)/AC_(0)_] × 100 (9)

where AC_(0)_ is the absorbance of the control at t = 0 min and AA_(t)_ is the absorbance of the antioxidant at t = 30 min. All measurements were conducted in triplicate, and the results are expressed as IC_50_ values in µg volatile compounds per mL hydrosol.

### 2.7. Antimicrobial Activity of Hydrosols

#### 2.7.1. Microdilution Assay: Minimum Inhibitory Concentration and Minimum Bactericidal Concentration

The minimum inhibitory concentration (MIC) and minimum bactericidal concentration (MBC) of the LnHYs were determined against five bacterial species: *Staphylococcus aureus* ATCC 25923, *Streptococcus pyogenes* (clinical isolate), *Escherichia coli* ATCC 25922, *Pseudomonas aeruginosa* ATCC 27853, and *Candida albicans* (clinical isolate). The standard microdilution technique in Mueller–Hinton broth (MHB) was used, following the original method described by Ericsson and Sherris in 1971 [25] with modifications under similar conditions [26]. Each hydrosol at its full concentration was used as the stock solution for the microdilution assay. Two-fold serial dilutions of the stock solutions were prepared in MHB to achieve final concentrations ranging from 100% to 3.06%. A volume of 100 μL of each diluted hydrosol was mixed with an equal volume of bacterial suspension. The assay included positive controls (broth with inoculum) and negative controls (broth without inoculum). Plates were incubated at 37 °C for 24 h with shaking at 120 rpm (Unimax 1010; Heidolph Instruments GmbH & Co. KG, Schwabach, Germany). MICs were defined as the lowest concentrations of hydrosol that prevented visible bacterial growth compared to control wells after 24 h of incubation. MBC values were determined by inoculating aliquots from the MIC assay wells onto Mueller–Hinton agar (MHA) plates, followed by further incubation for 18–24 h. MBC was defined as the lowest concentration of hydrosol that resulted in ≥99% bacterial death. Meropenem (for Gram-negative bacteria) and vancomycin (for Gram-positive bacteria) were used as positive controls for growth inhibition. The final antibiotic concentrations tested in the assay ranged from 0.0015 to 3.84 mg/L for both antibiotics.

Furthermore, the standard MIC/MBC method was improved by spectrophotometric determination of optical density at 600 nm due to visibly reduced turbidity and bacterial aggregation in the treated bacterial suspension after incubation. The method for determining growth inhibition by spectrophotometric optical density measurement was described by Beal in 2020 [27] and by Mira in 2022 [28].

#### 2.7.2. Determination of Bacterial Viability in Hydrosols

The bacterial suspension was prepared in Mueller–Hinton broth (MHB), and the concentration was standardised spectrophotometrically (OD600~1) to 10^9^ CFU/mL. This suspension was further serially diluted ten-fold to achieve a target concentration of 10^6^ CFU/mL. Aliquots of 2 mL of the bacterial suspension were transferred into sterile safety-lock tubes and centrifuged at 1190× *g* (r = 87 mm, Universal 320R, Hettich, Tuttlingen, Germany) for 10 min to pellet the bacterial cells. Following centrifugation, the supernatant was discarded, and the bacterial pellet was resuspended in hydrosol. The suspensions in hydrosol were incubated at 37 °C for 24 h. After incubation, 100 μL suspension was transferred onto the culture medium and spread evenly using a sterile plastic L-shaped spreader. The plates were then incubated again at 37 °C for 24 h.

### 2.8. Cell Cultures

Human epidermal keratinocyte cells (HaCaTs, CLS Cell Lines Service) were cultured in Dulbecco’s Modified Eagle’s Medium (DMEM, PAN-Biotech, Aidenbach, Germany) and supplemented with 1.0% antibiotics (100 U/mL penicillin and 100 μg/mL streptomycin, Sigma-Aldrich) and 10% foetal bovine serum (FBS, PAN-Biotech, Aidenbach, Germany) at 37 °C in 5% CO_2_ atmosphere in a humidified incubator.

### 2.9. Cell Viability Assay (XTT Assay)

The cytotoxicity of LnHYs was tested using the colorimetric method (XTT method) [16], which is based on the reduction of the yellow tetrazolium salt to an orange formazan dye by the enzyme dehydrogenase in metabolically active cells. The conversion occurs only in viable cells; therefore, the amount of formazan produced is proportional to the number of living cells in the sample, and formazan staining is quantified spectrophotometrically at 450 nm. To assess the suitability of the examined LnHYs for use on epidermal cells, a cytotoxicity test was performed on the human keratinocyte cell line (HaCaT).

HaCaT cells were seeded into 96-well plates at a density of 5 × 10^3^ cells per well in DMEM supplemented with 10% FBS and incubated for 24 h to allow cell attachment. Following incubation, the adherent cells were treated with varying concentrations of LnHYs (250–500 µg volatiles/mL hydrosol) in culture medium. The highest concentration (500 µg/mL; corresponding to 50 µL hydrosol) represents the maximum concentration permitted by the method. Cells were exposed to the treatment for 24 h. After the exposure period, the treatment medium was removed and replaced with 150 µL phenol red-free DMEM and 50 µL XTT detection solution, followed by an additional 3 h incubation at 37 °C to allow colorimetric development.

The water-soluble bright orange formazan dye formed in the assay was quantified by measuring absorbance at 450 nm using a microplate reader (BioTek Elx808, Winooski, VT, USA) [29,30,31].

An XTT (2,3-bis(2-methoxy-4-nitro-5-sulfophenyl)-2H-tetrazolium5-carboxanilide) Cell Viability Kit (#9095, Cell Signaling Technologies (Beverly, MA, USA)) was used to determine the effect of LnHYs on HaCaT cell viability.

### 2.10. Wound-Healing Assay (Scratch Assay)

The scratch assay was performed using the HaCaT cell line. Adherent cells grown in wells in a single layer, after inflicted mechanical injury, were treated with the examined LnHYs, and the closure of mechanical damage was monitored using an inverted microscope. By comparing the healing speed of treated and untreated cells, the impact of the tested LnHYs on wound healing was evaluated [16].

HaCaT cells were cultured in Dulbecco’s Modified Eagle’s Medium (DMEM) supplemented with 10% foetal bovine serum (FBS) and a 1% antibiotic–antimycotic solution containing penicillin, streptomycin, and amphotericin B. Cells were seeded at a density of 1 × 10^5^ cells per well in standard six-well plates and incubated at 37 °C in a humidified atmosphere with 5% CO_2_ until reaching 80–90% confluence.

Prior to the experiment, cells were starved for 24 h in serum-free DMEM to inhibit proliferation. A straight scratch was created on the monolayer of the HaCaT cells using a sterile 200 μL pipette tip, generating a cell-free area. Following the scratch, the culture medium was removed, and the cell monolayer was gently washed with Dulbecco′s Phosphate-Buffered Saline to eliminate detached cells and debris. Fresh serum-free medium containing 500 μL LnHY was then added, and the cells were incubated for 48 h.

The scratched area was photographed immediately after wounding (0 h) and subsequently at 24 h and 48 h using an inverted microscope (Olympus IX73, Olympus, Tokyo, Japan) equipped with a digital camera at 100× magnification. The total wound area was analysed using ImageJ software 1.54d (Java 1.8.0_345) (NIH, Bethesda, MD, USA).

Wound closure was monitored at 0 h, 24 h, and 48 h post-injury. Negative control wells received serum-free growth medium without LnHYs.

Wound closure was quantified and expressed as a percentage of the initial wound area (at 0 h) calculated using the following Equation (10):

Wound healing rate = (A_0_ − A_t_)/A_0_ × 100 (10)

where A_0_ is the scratch area at time 0 and A_t_ is the corresponding scratch area at 24 or 48 h. At least three independent experiments were performed, using three wells for each treatment.

### 2.11. Statistical Analysis

All experiments were performed in triplicate. Data from each experiment were statistically analysed using the R programming environment and Statistica 14.0 (TIBCO Software Inc., Palo Alto, CA, USA). One-way ANOVA was applied, followed by Tukey’s post hoc test for multiple comparisons, with a significance level set at *p* < 0.05. Principal Component Analysis (PCA) was performed for the polyphenolic substances (total polyphenols, tannins, total flavonoids, and total phenolic acids) in bay laurel leaves.

## 3. Results

### 3.1. Extraction and Identification of Volatile Components in Hydrosols of L. nobilis Leaf (LnHYs)

*L. nobilis* hydrosols (LnHYs) were obtained from three selected Croatian laurel localities by microwave-assisted extraction (MAE). The volatile components of the LnHYs were analysed using gas chromatography–mass spectrometry (GC-MS).

#### LnHY Composition Obtained by MAE

A comparative analysis of volatiles in the LnHYs based on their geographical origin is presented in Table 1, and the results are expressed as the relative amounts of each compound (percentage).

In all examined LnHY samples, more than 98% of the total volatile compounds were identified, with 1,8-cineole as the dominant compound in hydrosols collected from all three locations (ranging from 52.25% in the south to 81.89% in the north).

γ-Terpinene was the second most important component of the hydrosols from all locations, with the percentage of identification decreasing from north to south. In general, oxygenated monoterpenes were the main class in the composition of LnHYs from all examined localities.

A special feature is the class of monoterpene hydrocarbons, which were identified in hydrosols from the south at a proportion of 30.63% but were not detected in the north (Table 1).

Interestingly, the hydrosol derived from south exhibited notable differences in the composition of certain components, particularly in the relative amounts of α-pinene, E-caryophyllene, and methyl eugenol. These differences correspond to three major volatile compound classes: monoterpene hydrocarbons, sesquiterpene hydrocarbons, and phenolic compounds.

Notably, the phenolic compound methyl eugenol was detected in all three LnHYs, with the highest concentration observed in the south sample, indicating a potentially greater abundance of phenolic compounds in this region.

**Figure 1:**
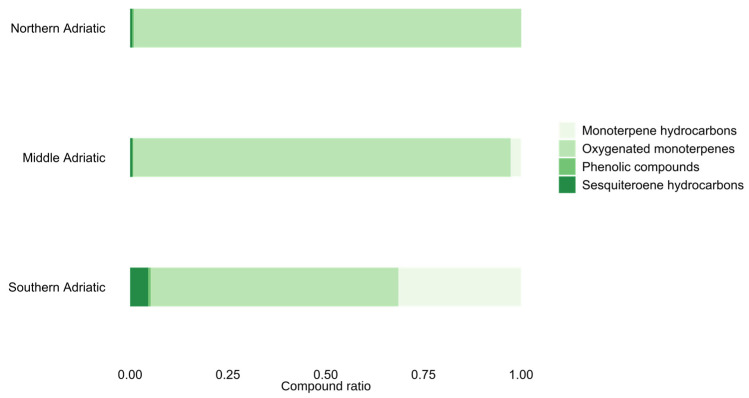
Distribution of compound ratios across three Adriatic regions.

The distribution of compound ratios across the Adriatic regions is shown in Figure 1. The stacked bar plot shows the proportional distribution of chemical compound groups in samples collected from the three Adriatic regions: northern, middle, and south. Compound ratios are normalised and plotted on the x-axis, while regions are shown on the y-axis. The bars are stacked to visualise the relative contribution of each compound. Namely, variations in compound ratios across regions suggest potential environmental or geographical influences on compound composition.

In general, the primary volatile components identified in the examined LnHYs include 1,8-cineole (52.25–81.89%), γ-terpinene (6.12–9.92%), terpinen-4-ol (1.77–2.84%), and E-caryophyllene (0.21–3.95%). α-Pinene was determined only in the middle and south samples (2.68% and 14.27%, respectively), while sabinene and β-phellandrene were exclusively characteristic of the south samples (8.39% and 8.01%, respectively).

### 3.2. Physical and Chemical Parameters of LnHYs

In this study, the relative density (*dr*), refractive index (*n*), pH, turbidity, acid value (*AV*), and essential oil content of LnHYs from three Croatian coastal regions were investigated to assess the purity and quality parameters of the hydrosols. The obtained values are presented in Table 2.

According to the available quality requirements, all LnHYs were of adequate quality, with no significant differences in the measured physical and chemical values between the investigated *L. nobilis* hydrosols.

### 3.3. Polyphenolic Substances in Bay Leaves

Plant polyphenols are an important group of bioactive compounds. They exhibit numerous biological activities, including antioxidant, anti-inflammatory, antitumor, and neuroprotective effects [33].

In this study, a polyphenol analysis was conducted on laurel leaf samples from the three locations using three different spectrophotometric methods: FCR assay (total polyphenol and tannin analysis); F-Al assay (total flavonoid analysis); and TPA assay (total phenolic acid analysis).

The findings (Table 3) indicate that the laurel leaf samples from the north region exhibited the highest levels of total polyphenols (TP) and tannins (T), measuring 7.44% and 3.20%, respectively. In contrast, the total flavonoid (TF) content was consistent across all examined regions (0.44–0.49%), and total phenolic acids (TPA) varied only slightly, with concentrations ranging from 2.21% to 2.73% (expressed as rosmarinic acid) and 4.12 to 5.09% (expressed as chlorogenic acid).

**Figure 2:**
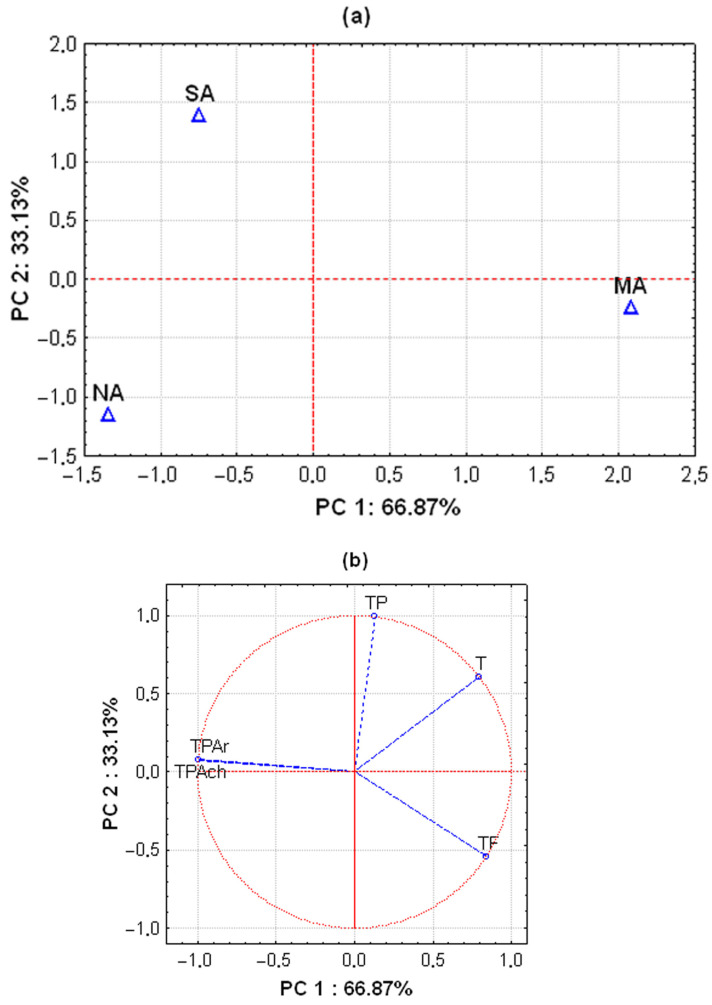
PCA analyses of polyphenolic substances in bay laurel leaves from north (NA), middle (MA), and south (SA) Adriatic regions (**a**). PCA loading plots of the polyphenolic substances from the first (PC 1) and the second (PC 2) principal components (**b**). The area inside the circle represents the region of valid loadings. TPs, total polyphenols; Ts, tannins; TFs, total flavonoids; TPArs, total phenolic acids expressed as rosmarinic acid; TPAchs, total phenolic acids expressed as chlorogenic acid.

Statistical analysis (PCA) was carried out for the polyphenolic substances in the laurel leaves. Principal component (PC) 1 explained 66.87%, while PC 2 explained 33.13% of the variance and distinguished the three locations from each other (Figure 2a). The south Adriatic is characterised by a higher TP content and intermediate TPAr and TPAch content. The north Adriatic is characterised by a higher TPAr and TPAch content, while the middle Adriatic is characterised by a higher T and TF content. The PCA loading plots of the polyphenolic substances from the first and second principal components are presented in Figure 2b.

### 3.4. Antioxidant Capacity of LnHYs

#### ORAC and DPPH Activity

The antioxidant capacity of the LnHYs was tested by the ORAC and DPPH methods, and the results are shown in Table 4.

All tested hydrosol extracts showed similar activity in both methods. The *L. nobilis* from the northern Adriatic region had slightly higher activity in the ORAC method, with a result of 1232.56 ± 121.81 µmol TE/g volatiles. In the case of DPPH, the sample from the middle Adriatic showed higher activity, with a result of 557.63 ± 13.66 µg of volatiles/mL of LnHYs.

### 3.5. Antimicrobial Activity of LnHYs

The antibacterial activity of the LnHYs from coastal Adriatic regions was tested on four microbial strains (two Gram-positive and two Gram-negative bacteria) and one fungal strain. The tested organisms were *Staphylococcus aureus* (ATCC 25923) and *Streptococcus pyogenes* (clinical isolate), as representatives of Gram-positive bacteria commonly associated with human epidermis and dermal infections, and *Escherichia coli* (ATCC 25922) and *Pseudomonas aeruginosa* (ATCC 27853), which are characteristic Gram-negative bacteria. Additionally, *Candida albicans* (clinical isolate) was chosen as a representative fungal strain commonly found in dermal environments [6,11].

#### 3.5.1. Standard MIC/MBC Testing

The LnHYs did not exhibit inhibitory properties in standard MIC/MBC testing (Table 5). MBC was not determined, and no minimum inhibitory concentration (MIC) was observed for most of the tested organisms. However, the minimum concentration required to inhibit the growth of *S. pyogenes* in this study was determined to be 50%. The positive control for *C. albicans* was not included.

**Figure 3:**
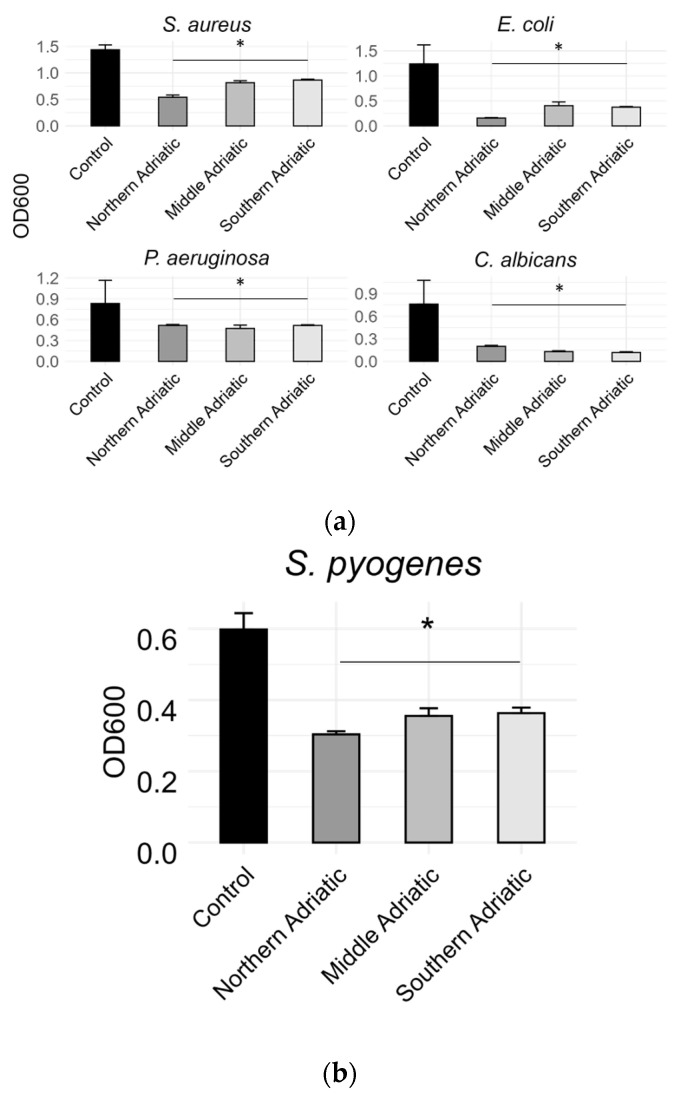
(**a**) Optical density measurements for three bacterial and one fungal strain treated with LnHYs from three Adriatic regions. The control group consisted of bacterial suspensions in Mueller–Hinton broth and fungal suspensions in Sabouraud broth, while the treatment group was prepared in a 1:1 (*v:v*) mixture of LnHYs and Mueller–Hinton broth or Sabouraud broth, respectively. (**b**) Optical density measurements for *S. pyogenes* treated with LnHYs. The control group consisted of bacterial suspensions in Mueller–Hinton broth, while the treatment group was prepared in a 1:4 (*v:v*) mixture of LnHYs and Mueller–Hinton broth. Results are expressed as mean values (*n* = 6) with one standard deviation. Asterisks (*) denote statistically significant differences between the hydrosol-treated groups and the control, determined using the nonparametric Mann–Whitney U test at a significance level of *p* < 0.05.

The minimum inhibitory concentration of the hydrosol for most bacterial strains was not determined, but a decrease in turbidity was observed with the addition of 50% hydrosol. Therefore, differences in optical density were measured and are presented in Figure 3.

Although the standardised method for determining the MIC did not yield conclusive results, apart from a 50% LnHY concentration for *S. pyogenes*, visible turbidity in the well and bacterial aggregation at the bottom indicated bacterial growth inhibition following hydrosol treatment. Due to this apparent growth inhibition, bacteria were subsequently treated with subinhibitory doses of hydrosol: 50% and 25% for *S. pyogenes*. Optical density was then measured using a spectrophotometer. The results showed a lower level of inhibition compared to the control group (bacteria in Mueller–Hinton broth), with bacterial growth being significantly inhibited. The mean log fold change was 1.26, which, when converted to CFU/mL using the formula provided by Deriase and El-Gendy [34], corresponds to a 5-log fold change.

#### 3.5.2. Bacterial Viability in LnHYs

After 24 h of incubation, none of the tested microorganisms exhibited growth in the undiluted LnHY, demonstrating its inhibitory potential against all evaluated strains, as shown in Table 6.

### 3.6. Cytotoxic Activity of LnHYs

The treatment of HaCaT human keratinocyte cells with LnHYs from three coastal Adriatic regions for 24 h resulted in no cytotoxic activity (Table 7).

### 3.7. Wound-Healing Rate of LnHYs

**Figure 4:**
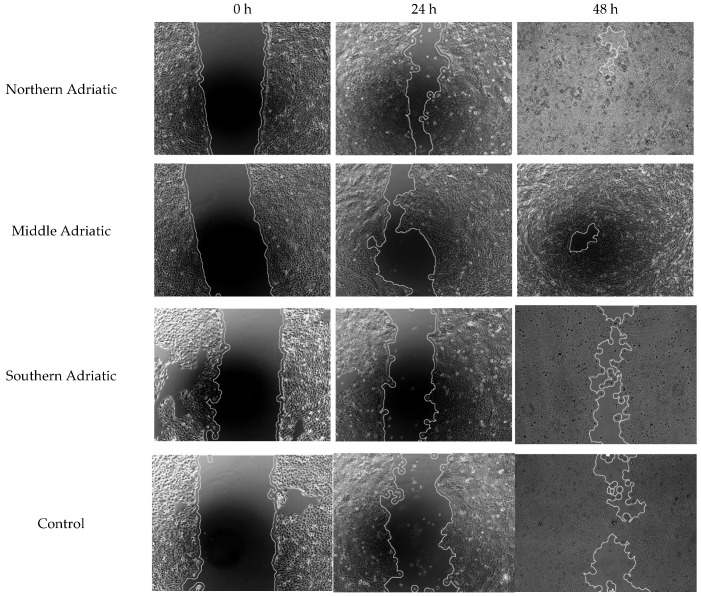
Microphotographs of wound-healing assay at timepoint 0 h, 24 h, and 48 h. Microscope magnification and scale bar is identical for all subfigures (400×).

Adherent HaCaT cell monolayers were treated with LnHYs obtained from three coastal regions of Croatia at a concentration of 400 µg volatiles/mL. The results are presented in Table 8 and Figure 4.

*L. nobilis* hydrosol from the middle Adriatic showed a mildly positive but statistically insignificant effect in promoting gap closure compared with untreated cells.

## 4. Discussion

This study provides a comprehensive analysis of bay laurel hydrosols (LnHYs) from three Adriatic regions of Croatia, including isolation methods, chemical composition, qualitative parameters, and the wide range of biological effects.

### 4.1. Phytochemical Analysis

Although often overlooked and treated as by-products of essential oil (EO) distillation, hydrosols (HYs) are garnering increasing attention for their potential applications in cosmetics and dermatology, particularly due to their milder properties compared to concentrated EOs. Despite significantly fewer studies examining HYs compared to EOs, there is growing evidence indicating that these aqueous distillates contain valuable bioactive compounds worthy of further scientific investigation [6].

The present study highlights variations in the volatile profiles of *Laurus nobilis* hydrosols (LnHYs) obtained from geographically distinct regions along the Adriatic coast. In all samples, 1,8-cineole emerged as the dominant compound, with the highest concentration (81.89%) observed in the north Adriatic—consistent with prior research identifying it as a major constituent in bay laurel leaves from various localities [3].

The second most abundant compound, γ-terpinene, exhibited a clear geographical trend, decreasing in concentration from north to south. This pattern may reflect the influence of environmental factors such as temperature and light intensity on monoterpene biosynthesis. Additionally, the south Adriatic sample was notable for its elevated levels of monoterpene hydrocarbons (30.63%), absent in the north region, possibly as a result of abiotic stresses affecting plant metabolism such as drought and salinity [35]. Compounds such as α-pinene, E-caryophyllene, and methyl eugenol were also more abundant in the southern samples. The higher methyl eugenol content, a phenolic compound with documented antimicrobial and antioxidant activity, is consistent with the literature that suggests that phenolic biosynthesis is sensitive to environmental conditions, including temperature and solar exposure [36]. These observations support the conclusion that geographical origin significantly shapes the chemical profiles of LnHYs, likely through complex interactions between genotype and local environmental stressors. This variability may, in turn, affect their biological activities and underscores the need for region-specific optimisation in both harvesting and extraction processes [37,38,39].

In terms of polyphenolic content, our results revealed moderate regional differences. The south samples exhibited slightly higher total polyphenol concentrations, while the middle region was particularly rich in tannins, compounds known for their antimicrobial and antioxidant properties. Flavonoid levels remained relatively consistent across the regions, suggesting that their biosynthesis may be less affected by geographical or climatic differences. Notably, total phenolic acids—especially rosmarinic and chlorogenic acid—were most concentrated in the north Adriatic samples, pointing to that region’s potential for producing antioxidant-rich plant material.

These findings align with previous studies demonstrating environmental influence on the phenolic composition of *L. nobilis*. For example, a comparative study analysing laurel leaves from Greece and Georgia revealed significant differences in polyphenol content between the two regions. The Greek samples had higher concentrations of free phenolic acids such as sinapic (607.7 μg/g), caffeic (586.1 μg/g), and ferulic acids (300.1 μg/g), whereas the Georgian samples were richer in conjugated phenolic acids, including sinapic (1513.9 μg/g) and caffeic acids (789.3 μg/g) [38]. Similarly, research comparing bay leaves collected from two Croatian coastal regions—Rijeka (north Adriatic) and Dubrovnik (south Adriatic)—found that the samples from Dubrovnik had a higher total polyphenol content (45.09 mg/g, 4.51%) compared to those from Rijeka (36.12 mg/g, 3.61%) [40].

In conclusion, the variations in polyphenolic profiles across different regions can be attributed to several environmental factors, which can influence the plant’s secondary metabolite production, leading to differences in polyphenol accumulation. Meteorological data collected for the harvest period (August 2022) of *L. nobilis* leaves included in our study support the hypothesis that environmental conditions influenced the observed phytochemical variability. According to official data from the Croatian Meteorological and Hydrological Service [41], all three sampling locations experienced a particularly dry and hot period, with mean temperatures ranging from 26.0 to 27.0 °C and numerous days exceeding 30 °C. These stress conditions are known to induce the synthesis of secondary metabolites, including phenolics and terpenes, as a defence mechanism, and may explain the regional differences observed in our study. An additional table with official meteorological data for the entire year of 2022 is included in the Supplementary Material (Supplementary S1).

Understanding these regional variations is crucial for optimising the use of laurel leaves in food, pharmaceutical, and cosmetic industries, where specific polyphenolic compounds may be desired for their antioxidant, antimicrobial, or therapeutic properties.

### 4.2. Quality Parameters

Due to their biological and organoleptic characteristics, hydrosols (HYs) are widely used in the food and cosmetic industries [42,43], serving as aqueous phases in various formulations. However, oxidative and polymerisation processes may compromise their quality and pharmacological activity. Thus, physicochemical parameters can support the quality control of commercial products. While pharmacopoeias define standard values for essential oils (EOs) [19,22,44], no reference values are currently available for laurel EO or LnHY. Accordingly, the measured parameters were compared to data from similar specimens [18,45].

Relative density (*dr*) correlates with the chemical composition of EOs; oils rich in terpenes typically have values below 0.900, while those with aromatic compounds exceed 1.000. In our study, the *dr* of LnHYs ranged from 0.962 to 1.021, values consistent with the aqueous nature of these distillates and with previously reported data [18]. The refractive index (*n*) is a simple indicator of the purity and quality of essential and fatty oils [22]. In our study, the *n* of LnHYs (1.333–1.334) aligned closely with water and is consistent with other studies on hydrosols (1.341–1.361) [18,22]. Turbidity, an indicator of water and HY quality, remained within WHO-acceptable limits (<5 NTU), ranging from 0.74 to 3.41, and were in accordance with the quality requirements [46,47]. The pH of hydrosols indicates the acidic or alkaline contamination caused by degradation of the active ingredients. Therefore, pH is applied to determine HY stability and purity upon storage [22]. The pH of HYs ranges from 4.5 to 5.5, depending on the herbal drug. HYs with a lower pH have a better inhibitory effect on bacteria [48,49]. The pH values of the LnHYs (3.62–4.09) were comparable to those of immortelle HY (3.5–3.8) [50], indicating acceptable purity and quality. The acid value, an indicator of degradation, was very low across all samples (0.0088–0.0096 mg KOH/g), as expected for hydrosols with low volatile content [51,52]. Finally, the EO content in the LnHYs was 0.007–0.014%, reflecting expected values due to the hydrophilic nature of HYs and geographical variations. Despite their low concentration, the presence of EO components in HYs contributes to their overall biological profile [53].

### 4.3. Biological Effects

While *L. nobilis* has been extensively studied for its EO and polyphenol-rich extracts [7,8,9,10,11], the biological effects of its hydrosols remain largely unexplored [12]. The present study provides novel data in this regard.

All three LnHYs demonstrated high antioxidant capacity, highlighting their potential as natural antioxidants.

Importantly, none of the hydrosols showed cytotoxic effects on human keratinocyte (HaCaT) cells, even at the highest tested concentration (500 µg volatiles/mL). These findings provide preliminary support for the safe use of hydrosols in dermatological and cosmetic applications and align with the general consensus that hydrosols are well-tolerated due to their low concentrations of active compounds. The observed preservation of cell viability, even at maximum tested concentrations, may be attributed to the relatively low levels of bioactive constituents typically present in hydrosols as compared to essential oils. This is consistent with the overarching goal of the study—to explore the biological potential of hydrosols as secondary products generated during essential oil distillation. To gain a more comprehensive understanding of the effects of LnHYs on skin cells, future studies would benefit from incorporating extended exposure and employing additional assays to evaluate a broader spectrum of functional cellular responses.

Regarding wound-healing potential, our study revealed a neutral to mildly positive effect. Although the LnHYs exhibited a slight enhancement in wound-healing rate, the overall impact remains modest. However, we believe that these findings are still valuable, as this is the first time such an evaluation of these specific hydrosols has been conducted and published. The absence of cytotoxic effects is relevant, particularly in the context of potential safe topical applications.

The antimicrobial activity of LnHYs, assessed using standardised microbiological methods, was not confirmed, except against *S. pyogenes*. These findings align with previous research conducted by Ovidi et al. [6]. However, during the MIC/MBC testing, all bacterial samples exhibited reduced turbidity, prompting further investigation into the antimicrobial potential of LnHYs. The limited microbiological activity observed is likely due to the high water content in hydrosols, leading to low concentrations of antimicrobial terpenes. To address this limitation, we modified our methodology by incubating bacteria in undiluted hydrosols and further evaluating bacteriostatic activity through optical density measurements of bacterial suspensions treated with LnHYs. This adjustment revealed a bactericidal effect of the hydrosols compared to water, providing new insights into their potential antimicrobial properties. In conclusion, the hydrosols showed weak antimicrobial activity, which was supported by findings obtained using an additional, non-standard analytical method.

Our results indicate that pure hydrosols may be particularly suited for topical use given that their strongest effect was observed against *S. pyogenes*, a common skin pathogen. Unlike EOs, which require dilution due to their potency and potential irritancy, HYs are milder aqueous distillates with significantly lower concentrations of volatiles. Given their safety profile and mild biological activity, LnHYs may serve as promising ingredients in formulations targeting sensitive or infection-prone skin. Recent studies support this, confirming that hydrosols from other medicinal plants (e.g., *Thymus vulgaris* and *Lavandula officinalis*) are safe and effective even on compromised skin [54]. Since hydrosols are typically used undiluted in topical formulations, we believe that assessing their efficacy in this form is both justified and practically relevant.

Overall, our findings deepen the understanding of laurel hydrosol properties and the influence of geographical origin on its phytochemical composition, with particular emphasis on its antioxidant capacity, potential antimicrobial activity, and the safety of its topical application.

## 5. Conclusions

This study offers a comprehensive phytochemical and biological characterisation of *Laurus nobilis* hydrosols (LnHYs) produced by microwave-assisted extraction (MAE), an environmentally sustainable and efficient green technology. Detailed phytochemical analysis confirmed the consistent presence of volatile compounds and polyphenols across samples, with minor regional variation.

Biological testing demonstrated notable antioxidant capacity, weak antimicrobial activity, and neutral proliferative effects in a wound-healing model. These attributes, combined with the absence of cytotoxicity, show the potential of LnHYs for topical applications.

While these findings highlight the promise of LnHYs as biocompatible ingredients obtained through sustainable means, their dermatological potential remains to be additionally evaluated.

Future research should focus on long-term stability, formulation compatibility, and in vivo efficacy to fully validate their practical applications. This work thus lays a valuable foundation for the continued exploration and innovation of botanical hydrosols in modern dermopharmaceutical science.

## Figures and Tables

**Table 1:** Chemical composition of LnHYs from three coastal regions of Croatia.

			Northern Adriatic	Middle Adriatic	Southern Adriatic
Component	RI^a^	RI^b^	HY ± SD (%)	HY ± SD (%)	HY ± SD (%)
Monoterpene hydrocarbons			**-**	**2.68**	**30.63**
*α*-Pinene *	935	1017	-	2.68 ± 0.10	14.27 ± 0.01
Sabinene	971	1121	-	-	8.39 ± 0.01
*β*-Phellandrene	1002	1195	-	-	8.01 ± 0.01
Oxygenated monoterpenes			**97.00**	**94.09**	**61.72**
*1,8*-Cineole	1026	1210	81.89 ± 0.01	80.73 ± 0.01	52.25 ± 0.01
γ-Terpinene	1057	1225	9.92 ± 0.01	8.47 ± 0.01	6.12 ± 0.01
Linalool	1095	1506	0.35 ± 0.01	0.66 ± 0.03	0.12 ± 0.01
*allo*-Ocimene	1128	1390	0.21 ± 0.01	-	0.11 ± 0.15
Terpinen-4-ol	1174	1686	2.84 ± 0.01	2.32 ± 0.01	1.77 ± 0.01
*α*-Terpineol	1184	1660	1.31 ± 0.01	1.52 ± 0.15	0.91 ± 0.05
Bornyl acetate	1285	1570	0.18 ± 0.01	0.17 ± 0.01	0.21 ± 0.01
α-Terpinyl acetate	1349	1689	0.30 ± 0.10	0.22 ± 0.01	0.23 ± 0.01
Sesquiterpene hydrocarbons			**0.55**	**0.58**	**4.49**
*E*-Caryophyllene *	1424	1585	0.55 ± 0.01	0.21 ± 0.01	3.95 ± 0.01
*allo*-Aromadendrene	1465	1662	-	0.37 ± 0.01	-
Germacrene D	1481	1692	-	-	0.42 ± 0.01
*δ*-Cadinene	1517	1745	-	-	0.12 ± 0.01
Oxygenated sesquiterpenes			**0.77**	**0.57**	**0.92**
Spathulenol	1577	2101	-	-	-
Caryophyllene oxide *	1581	1955	0.77 ± 0.01	0.57 ± 0.01	0.92 ± 0.01
Phenolic compounds			**0.37**	**0.18**	**0.62**
Thymol *	1289	2154	**-**	**-**	0.14 ± 0.01
Methyl eugenol	1403	2005	0.37 ± 0.01	0.18 ± 0.01	0.48 ± 0.01
**Total identification (%)**			**98.69**	**98.10**	**98.42**

Retention indices (RIs) were determined relative to a series of n-alkanes (C8–C40) on capillary columns VF-5 ms (RI^a^) and CPWax 52 (RI^b^). Identification method: RI, comparison of RIs with those in a self-generated library reported in the literature [32] and/or with authentic samples; comparison of mass spectra with those in the NIST02 and Wiley 9 mass spectral libraries; * co-injection with reference compounds; -, not identified; SD, standard deviation of triplicate analysis.

**Table 2:** Physical and chemical parameters in quality control of LnHYs [22].

Parameters	Northern Adriatic	Middle Adriatic	Southern Adriatic
*dr* (at 20 °C)	0.962 ± 0.003	0.986 ± 0.004	1.021 ± 0.002
*n*/20 °C	1.334 ± 0.002	1.333 ± 0.001	1.333 ± 0.001
pH/20 °C	3.62 ± 0.04	3.82 ± 0.03	4.09 ± 0.02
turbidity (NTU)	3.41 ± 0.04	0.74 ± 0.02	1.02 ± 0.05
*AV* (mg KOH/g)	0.0096 ± 0.0003	0.0092 ± 0.0003	0.0088 ± 0.0003
essential oil content (%)	0.0076 ± 0.0004	0.014 ± 0.004	0.0069 ± 0.0004

**Table 3:** Quantification of total polyphenols (TPs), tannins (Ts), flavonoids (TFs), and phenolic acids (TPAs) in bay laurel leaf samples.

Specimen	TP	T	TF	TPAr *	TPAch **
	(% DW)	(% DW)	(% DW)	(% DW)	(% DW)
Northern Adriatic	6.57 ± 0.43	2.63 ± 0.39	0.46 ± 0.03	2.73 ± 0.32	5.09 ± 0.61
Middle Adriatic	6.98 ± 0.82	3.38 ± 0.16	0.49 ± 0.24	2.21 ± 0.78	4.12 ± 1.46
Southern Adriatic	7.44 ± 0.80	3.20 ± 0.74	0.44 ± 0.13	2.68 ± 0.41	5.00 ± 0.77

Note: DW—dry weight; * TPAr—total phenolic acid content expressed as rosmarinic acid; ** TPAch—total phenolic acid content expressed as chlorogenic acid.

**Table 4:** Antioxidant activity of hydrosols from three Adriatic regions determined using the ORAC and DPPH methods.

Specimen	ORAC ± SD	DPPH ± SD
Northern Adriatic	1232.56 ± 121.81	502.23 ± 12.46
Middle Adriatic	1222.85 ± 67.46	557.63 ± 13.66
Southern Adriatic	1157.38 ± 106.14	518.53 ± 26.27

ORAC, oxygen radical absorbance capacity, results for hydrosols expressed as µmol Trolox equivalents (TE) per g volatile compounds extracted in hydrosol. DPPH results for hydrosols expressed as IC50 in µg volatiles/mL hydrosol, SD = standard deviation of triplicate analysis.

**Table 5:** Minimal inhibitory concentration of LnHYs from three Croatian coastal regions.

	*L. nobilis* Hydrosols MIC (%)			Control MIC (μg/mL)	
Test Strains	North	Middle	South	Meropenem	Vancomycin
*Staphylococcus aureus* (ATCC 25923)	>50%	>50%	>50%	ND	1
*Streptococcus pyogenes* (clinical isolate)	50%	50%	50%	0.016	ND
*Escherichia coli* (ATCC 25922)	>50%	>50%	>50%	0.5	ND
*Pseudomonas aeruginosa* (ATCC 27853)	>50%	>50%	>50%	ND	0.5
*Candida albicans* (clinical isolate)	>50%	>50%	>50%	ND	ND

ND—not determined.

**Table 6:** Bacterial viability in different LnHYs from three Croatian coastal regions.

Bacteria	*L. nobilis* Hydrosols		
	North	Middle	South
*Staphylococcus aureus* (ATCC 25923)	-	-	-
*Streptococcus pyogenes* (clinical isolate)	-	-	-
*Escherichia coli* (ATCC 25922)	-	-	-
*Pseudomonas aeruginosa* (ATCC 27853)	-	-	-
*Candida albicans* (clinical isolate)	-	-	-

**Table 7:** Inhibitory potential of total hydrosol samples from three Croatian coastal regions.

Concentration	*L. nobilis* Hydrosols		
	North	Middle	South
250 µg/mL	NA	NA	NA
500 µg/mL	NA	NA	NA

The concentration of hydrosol used in the cell viability assay is expressed as µg volatile compounds per mL hydrosol; NA—no activity.

**Table 8:** Wound-healing rate at 24 h and 48 h after treatment with *L. nobilis* hydrosols from three locations; *p* value was calculated in comparison with untreated cells.

Samples	% Wound-Healing Rate 24 h	% Wound-Healing Rate 48 h	*p* Value
North Adriatic	46.28 ± 26.47	73.71 ± 17.88	0.65
Middle Adriatic	52.30 ± 17.70	86.25 ± 46.48	0.16
Southern Adriatic	37.93 ± 17.05	68.51 ± 23.78	0.08
Control	45.34 ± 19.11	83.38 ± 21.70	

Statistical significance level was set at *p* < 0.05.

## Data Availability

The original contributions presented in this study are primarily included in the article, while additional data are available from the corresponding author upon request due to planned future analyses of the dataset.

## Supplementary Materials

The following supporting information can be downloaded at: https://www.mdpi.com/article/10.3390/antiox14060688/s1.
