# Supplementary material for: Chemical Composition, Quality, and Bioactivity of Laurus nobilis L. Hydrosols from the Adriatic Regions of Croatia: Implications for Dermatological Applications
Source: Antioxidants (Basel). 2025 Jun 5;14(6):688. doi: 10.3390/antiox14060688 (PMC12189544; doi:10.3390/antiox14060688)
Supplement: Supplementary file 1 [file antioxidants-14-00688-s001.zip › antioxidants-3594417-supplementary.pdf]

Suppl. S1. Climatic indicators for selected Adriatic locations (Lovran, Tisno, Korčula) according to data from the Croatian Meteorological and Hydrological Service (2022)

LOVRAN - Average monthly air temperature °C

YEAR	I	II	III	IV	V	VI	VII	VIII	IX	X	XI	XII	AVG.
2022	6.5	8.3	9.2	12.3	19.9	25.3	27.0	26.0	19.1	17.9	12.0	9.2	16.1

LOVRAN - Number of hot days Tmax ≥ 30 °C

YEAR	I	II	III	IV	V	VI	VII	VIII	IX	X	XI	XII	SUM
2022	0	0	0	0	1	20	24	23	2	0	0	0	70

LOVRAN - Average monthly and annual precipitation amounts (mm)

YEAR	I	II	III	IV	V	VI	VII	VIII	IX	X	XI	XII	SUM
2022	34.2	84.8	36.6	116.6	52.5	27.4	48.8	23.5	592.6	67.2	147.4	335.5	1567.1

LOVRAN - Average monthly relative humidity (%)

YEAR	I	II	III	IV	V	VI	VII	VIII	IX	X	XI	XII	AVG.
2022	63	66	48	62	59	54	45	52	69	71	70	81	62

TISNO - Average monthly air temperature °C

YEAR	I	II	III	IV	V	VI	VII	VIII	IX	X	XI	XII	AVG.
2022	7.2	9.3	9.4	13.2	20.6	26.3	28.0	26.6	21.1	18.6	13.0	11.6	17.1

TISNO - Number of hot days Tmax ≥ 30 °C

YEAR	I	II	III	IV	V	VI	VII	VIII	IX	X	XI	XII	SUM
2022	0	0	0	0	3	23	28	27	4	0	0	0	85

TISNO - Average monthly and annual precipitation amounts (mm)

YEAR	I	II	III	IV	V	VI	VII	VIII	IX	X	XI	XII	SUM
2022	13.5	70.1	9.0	56.2	24.2	17.1	52.2	11.7	45.2	0.0	205.8	154.7	659.7

TISNO - Average monthly relative humidity (%)

YEAR	I	II	III	IV	V	VI	VII	VIII	IX	X	XI	XII	AVG.
2022	60	63	51	61	61	54	46	54	63	69	71	81	61

KORČULA - Average monthly air temperature °C													
**YEAR**	**I**	**II**	**III**	**IV**	**V**	**VI**	**VII**	**VIII**	**IX**	**X**	**XI**	**XII**	**AVG.**
**2022**	9.5	10.9	10.8	14.7	21.0	26.5	28.0	27.0	22.4	19.4	15.0	13.3	18.2

KORČULA - Number of hot days Tmax ≥ 30 °C													
**YEAR**	**I**	**II**	**III**	**IV**	**V**	**VI**	**VII**	**VIII**	**IX**	**X**	**XI**	**XII**	**SUM**
**2022**	0	0	0	0	0	20	24	20	0	0	0	0	64

KORČULA - Average monthly and annual precipitation amounts (mm)													
**YEAR**	**I**	**II**	**III**	**IV**	**V**	**VI**	**VII**	**VIII**	**IX**	**X**	**XI**	**XII**	**SUM**
**2022**	1.0	19.0	30.0	70.0	25.0	75.5	22.7	26.6	68.6	5.2	207.1	158.9	709.6

KORČULA - Average monthly relative humidity (%)													
**YEAR**	**I**	**II**	**III**	**IV**	**V**	**VI**	**VII**	**VIII**	**IX**	**X**	**XI**	**XII**	**AVG.**
**2022**	63	68	57	65	64	59	53	62	67	77	-	77	65
